# Cardiac and Liver Fibrosis Assessed by Multiparametric MRI in Patients with Fontan Circulation

**DOI:** 10.1007/s00246-024-03522-9

**Published:** 2024-05-21

**Authors:** Adriana Innocenzi, Isabela Rangel, Mariana Póvoa-Corrêa, Daniella Braz Parente, Renata Perez, Rosana Souza Rodrigues, Lúcia Tomoko Fukuyama, Julia Machado Barroso, Jaime Araújo Oliveira Neto, Andréa Silvestre de Sousa, Ronir Raggio Luiz, Rosa Célia Pimentel Barbosa, Gabriel Cordeiro Camargo, Renata Moll-Bernardes

**Affiliations:** 1 D’Or Institute for Research and Education (IDOR), Diniz Cordeiro, 30, Botafogo, Rio de Janeiro, RJ 22281-100 Brazil; 2 Instituto Nacional de Cardiologia, Rio de Janeiro, RJ Brazil; 3 Pro Criança Cardiaca, Rio de Janeiro, RJ Brazil; 4 Clínica Cardiológica Infantil, Rio de Janeiro, RJ Brazil; 5 Federal University of Rio de Janeiro (UFRJ), Macaé, RJ Brazil; 6 Federal University of Rio de Janeiro (UFRJ), Rio de Janeiro, RJ Brazil; 7 Evandro Chagas National Institute of Infectious Diseases, Oswaldo Cruz Foundation, Rio de Janeiro, Brazil

**Keywords:** Fontan, Fibrosis, T1 mapping, Extracellular volume, MRI, Fontan-associated liver disease

## Abstract

The abnormal hemodynamics in Fontan circulation due to persistently increased systemic venous pressure results in hepatic venous congestion and Fontan-associated liver disease. Combined assessment of cardiac and liver fibrosis and cardiac remodeling using multiparametric MRI in this context have not been fully explored. To evaluate cardiac and liver fibrosis and cardiac remodeling using multiparametric MRI in patients who have undergone Fontan procedures. Thirty-eight patients and 23 controls underwent cardiac and liver MRI examinations in a 3.0-T scanner. Mann–Whitney, Fisher exact test, and Spearman’s correlation were applied to evaluate myocardial volumes, function, native cardiac and liver T1 mapping, ECVs and liver stiffness. The mean native cardiac T1 value (*p* = 0.018), cardiac ECV (*p* < 0.001), liver native T1 (*p* < 0.001), liver ECV (*p* < 0.001), and liver stiffness (*p* < 0.001) were higher in patients than controls. The indexed end-diastolic volume (EDVi) correlated with the myocardial ECV (*r* = 0.356; *p* = 0.033), native liver T1 (*r* = 0.571; *p* < 0.001), and with liver stiffness (*r* = 0.391; *p* = 0.015). In addition, liver stiffness correlated with liver ECV (*r* = 0.361; *p* = 0.031) and native liver T1 (*r* = 0.458; *p* = 0.004). An association between cardiac remodeling and cardiac and liver fibrosis were found in this population. The usefulness of MRI to follow cardiac and liver involvement in these patients is critical to improve treatment strategies and to prevent the need for combined liver and heart transplantation.

## Introduction

Over the past decades, increased survival has been observed among individuals with single functional ventricles who undergo total cavopulmonary connection, known as the Fontan procedure [1–3]. With this procedure, the systemic venous return is connected directly to the pulmonary arteries, resulting in passive cavopulmonary flow with no pulmonary ventricular pumping.

This unique physiology usually leads to persistent chronic systemic venous congestion, reduced cardiac output, and chronic hypoxia affecting the heart and the liver. The systolic ventricular function may remain preserved for several years following this surgery [3], although it tends to decline over time. Up to 30–50% of adult patients with Fontan circulation are expected to develop circuit failure [4–7].

Cardiac magnetic resonance imaging (MRI) assesses morphological and functional parameters, such as the cavitary volume, ejection fraction (EF), cavopulmonary circuit, and pulmonary arteries. Cardiac MRI with late gadolinium enhancement (LGE) can be used to assess myocardial fibrosis, an important prognostic marker for various cardiac diseases.

However, the use of LGE may lead to the underestimation of interstitial myocardial fibrosis. New parameters for tissue characterization, including native T1 mapping and extracellular volume (ECV) calculation, enable the quantification of interstitial and diffuse myocardial fibrosis, with good correlation with histological findings [8–12].

The spectrum of Fontan-associated liver disease (FALD) presentations ranges from minor changes, such as hepatomegaly and hepatic vein dilation to more severe modifications indicative of advanced fibrosis, including a nodular liver texture and signs of portal hypertension. Although liver disease has been described in many patients who have undergone Fontan procedures, its associations with cardiac remodeling and myocardial fibrosis have not been fully explored.

Liver MRI assesses liver parenchyma, nodular enhancement, liver stiffness, and signs of chronic liver disease. The earliest abnormalities found in FALD involve hepatic congestion characterized by hepatic vein and sinusoidal dilation [13–15]. Although there are new MRI parameters to assess liver fibrosis, congestion, and stiffness [16, 17], these techniques have not been fully explored and there are no clear guidelines to study liver disease in patients with Fontan. Moreover, the association between liver and cardiac fibrosis is not clear.

The purpose of this study was to assess cardiac and liver MRI parameters, including myocardial dimensions and function, LGE, myocardial and liver ECV, and liver stiffness in patients with univentricular physiology who have undergone the Fontan procedure. Ventricular remodeling and volumetric overload could be early markers of diffuse interstitial fibrosis and be associated with liver congestion, which is one important mechanism of FALD progression.

## Materials and Methods

### Study Population

Consecutive outpatients with complex cardiac malformations and univentricular physiology who had undergone Fontan surgery and control subjects were enrolled prospectively in this cross-sectional observational study. All patients were aged ≥ 7 years and had clinical indications for routine imaging studies to follow the Fontan circulation. Patients with MRI contraindications, such as pacemakers, implantable cardio-defibrillators, claustrophobia, or gadolinium allergy were excluded. Control subjects had no symptoms related to cardiac or liver disease, had less than two risk factors for cardiovascular disease and had no signs of cardiac or liver disease on MRI.

Recruitment occurred between March 2019 and July 2023. The institutional review board and ethics committee of the D'Or Institute for Research and Education approved the study protocol (CAAE#70699417.7.3001.5272). All patients or guardians gave written informed consent to undergo the diagnostic procedures and to the publication of the study data.

### Data Collection

Data on the patients' demographic, anatomical, surgical, and clinical attributes were extracted from their medical records and included patient’s age at the time of Fontan procedure performance, surgical technique, type of Fontan, time since Fontan procedure performance, previous diagnosis of chronic liver disease, and New York Heart Association (NYHA) functional class.

### MRI Protocol

The study involved comprehensive cardiac and liver MRI evaluations. Patients underwent MRI examination with a 3.0-T scanner (MAGNETOM Prisma; Siemens Healthcare, Erlangen, Germany) without sedation or anesthesia. A 4-h fasting period was implemented to mitigate potential confounding factors in liver stiffness assessment, particularly the postprandial effect [18]. Peripheral blood samples were collected for hematocrit determination on the same day of the MRI examination.

During the examination (ca. 50 min), the patients were given intravenous infusions of gadolinium-DOTA (0.3 mmol/kg; Dotarem; Guerbet). For cardiac MRI, electrocardiographic (ECG) gated static and cine steady-state free precession techniques were used to assess cardiac morphology and function. Cine images were acquired in long- and short-axis planes with a gradient-echo [steady-state free precession (SSFP)] pulse sequence.

Cardiac T1 mapping was performed with a modified look-locker inversion recovery (MOLLI) sequence using single-shot SSFP readouts. The 5(3)3 MOLLI sequence design was used for pre-contrast (native) T1 mapping and post-contrast acquisition [19]. Mean native and post-contrast myocardial T1 values were acquired with single short-axis images at the mid-ventricular level taken before and ≥ 15 min after gadolinium injection. LGE images were obtained using a breath-hold segmented gradient-recalled echo inversion recovery procedure at ≥ 10 min after contrast injection. The inversion time was adjusted to null myocardial signals in the range of 230–350 ms, and images were acquired at every R-R interval when the heart rate was ≤ 75 bpm and at every other R-R interval when the heart rate was > 75 bpm.

For the liver examination, an axial turbo spin-echo sequence and coronal T2 half-Fourier single-shot and axial 3D T1-weighted volumetric interpolated apnea scans were performed before and after contrast administration. To assess inferior vena cava, we employed breath-hold contrast-enhanced magnetic resonance angiography with multiplanar reformatting, enabling measurements and 3D cross-sectional imaging perpendicular to the vessel wall [20–22]

Liver T1 mapping, the MOLLI recovery sequence, consisting of acquisitions of eight images at a fixed time of 5(3)5, was employed [16, 23].

The liver MRI 2D-gradient echo magnetic resonance elastography (MRE) was performed to assess liver stiffness. The sequence uses 2D gradient-recalled echo MRE acquire liver elasticity maps. The parameters were: TR: 25 ms; TE: 15.19 ms; flip angle: 12°; FOV: 380 mm; active driver frequency: 60 Hz; voxel size: 1.5 × 1.5 × 6 mm; slices: 4; acceleration factor: GRAPPA 2. To evaluate liver stiffness, a pneumatic active wave driver and a tube-connected and strap-secured passive driver were put on the right anterior chest wall at the level of xiphoid appendages. Generated shear waves at specific vibration frequency displaced liver tissue to make magnitude and phase images. The 4 slices were acquired in end-expiration breath holds. Following the creation of magnitude and phase images, an inversion algorithm within the MRI unit automatically processed raw data to generate additional images and maps [17, 18].

### MRI Analysis

Images were analyzed using multiplatform commercial software (OsiriX MD; Pixmeo) and Circle CVI42 (Circle Cardiovascular Imaging).

Cardiac chambers volume and dominant ventricle ejection fraction were obtained from cine images according to a standard protocol [24]. Myocardial pre- and post-contrast T1 properties were quantified directly from T1 mapping images. The mean signal intensity value was obtained from a region of interest (ROI) considering the entire compact myocardium of the dominant single ventricle. In addition, a 2-cm^2^ circular ROI was placed in the center of the main ventricular cavity on T1 maps to obtain pre- and post-contrast blood pool values for ECV calculation. The LGE mass in the myocardium was characterized using visual semiquantitative scores [25]. The extent of LGE was determined in each of the 17 myocardial segments on short-axis LGE images. The ECV was calculated using the hematocrit value to adjust for contrast volume distribution. Two experts with more than 8 years of experience in cardiac and liver MRI conducted the analyses.

Liver morphology, contour, signs of chronic liver disease, hepatic congestion, focal liver lesions, enlarged inferior vena cava, ascites and abdominal collateral veins were assessed. Hepatic congestion was characterized by T2-weighted images in the hepatic parenchyma and periportal regions, in combination with post-contrast heterogeneous enhancement pattern and large hepatic veins and inferior vena cava. Hepatomegaly and hepatic veins were assessed qualitatively. An enlarged vena cava was defined as having a z-score greater than two. The general morphology of the liver and the presence or absence of focal liver lesions were described.

For native and post-contrast liver T1 mapping, the region of interest was drawn at a depth of 1 cm from the liver surface, covering most of the liver parenchyma, while avoiding regions with artifacts and main vascular structures.

Elastography generated a color scale elastogram ranging from 0 to 8 kPa. Post-processing was automatically executed on the scanner MRI console, and mean liver stiffness was calculated as the average of ROIs drawn in each of the four liver slices, avoiding areas with large vessels, liver edge, fissures, and regions of ambiguous wave propagation within 95% confidence map.

### Statistical Analysis

Means and standard deviations or median and interquartile range were calculated for continuous variables. Categorical variables are described as proportions. The Kolmogorov–Smirnov test was used to examine variable distributions. Mann–Whitney and Fisher exact tests were used for between-group comparisons, as appropriate. Spearman’s analysis was used to examine correlations among cardiac and liver MRI parameters. For all tests, the significance level was set to *p* < 0.05. The data were analyzed using SPSS (version 21; IBM Corporation).

## Results

### Patient Characteristics

The study sample comprised 38 patients with Fontan circulation and 23 controls. From the Fontan group, 23 (60%) were female, and the average age was 16.7 (range 7–31) years, and the mean body mass index was 20.8 ± 4.0 kg/m^2^. All patients were NYHA functional class I. Fourteen patients with Fontan had previous complications, including stroke in four, protein-losing enteropathy in one, ventricular tachycardia in five and supraventricular tachycardia in four cases. The mean oxygen saturation was 92.6% ± 3.8%. The baseline characteristics of the patients and controls are shown in Table 1.

**Table 1:** Baseline characteristics of the patients (*n* = 38) and controls (*n* = 23)

	Fontan (*n* = 38)	Controls (*n* = 23)	*p*
Age, years	16.7 ± 5.3	55.8 ± 11.2	< 0.001
Female gender, *n* (%)	21 (55.3)	15 (65.2)	0.592
BMI, kg/m^2^	20.8 ± 4.0	27.6 ± 5.6	< 0.001
NYHA I, *n* (%)	38 (100)	23 (100)	1.0
Systemic hypertension, *n* (%)	0	2	
Protein-losing enteropathy, *n* (%)	1 (2.6)	0	
Hematocrit, %	45.9 ± 4.4	42.4 ± 3.0	0.001
Atrioventricular regurgitation^a^, *n* (%)	3 (7.9)	0	
Ventricular dysfunction^b^, *n* (%)	3 (7.9)	0	

Values are n (%) or mean ± standard deviation

*BMI* body mass index, *NYHA* New York Heart Association

^a^Moderate to severe regurgitation on echocardiography

^b^Moderate dysfunction on echocardiography

The main diagnoses were tricuspid atresia (*n* = 16), hypoplastic left heart syndrome (*n* = 6), pulmonary atresia (*n* = 4), double inlet left ventricle (*n* = 4), great vessel transposition (*n* = 4), congenitally corrected great vessel transposition (*n* = 2), unbalanced atrioventricular septal defect (*n* = 1), and tricuspid hypoplasia (*n* = 1). On echocardiography, 35 (92.1%) patients had no, or mild global ventricular dysfunction and 35 (92.1%) patients exhibited no or mild valvar regurgitation. The Fontan characteristics are listed in Table 2.

**Table 2:** Fontan characteristics

	Fontan (*n* = 38)
Post Fontan time, years	8.2 ± 6.7
Aortic clamping, min	73.4 ± 36.4
Fenestration at Fontan surgery, *n* (%)	14 (36.8)
Main diagnosis, *n* (%)	
Tricuspid atresia	16 (42.1)
Hypoplastic left heart syndrome	6 (15.8)
Pulmonary atresia	4 (10.5)
Double inlet left ventricle	4 (10.5)
Great vessel transposition	4 (10.5)
Congenitally corrected great vessel transposition	2 (5.3)
Unbalanced atrioventricular septal defect	1 (2.6)
Tricuspid hypoplasia	1 (2.6)
Fontan modality, *n* (%)	
Extracardiac	34 (89.5)
Lateral tunnel	2 (5.3)
Atriopulmonary connection	1 (2.6)
Intra-cardiac	1 (2.6)
Single ventricle morphology, *n* (%)	
Right	7 (18.4)
Left	26 (68.4)
Biventricular^a^	5 (13.2)

Values are *n* (%) or mean ± standard deviation

^a^Left and right balanced ventricles with large ventricular septal defect

### Previous Procedures

All patients had undergone partial cavopulmonary surgery, 24 (63%) patients had undergone Blalock-Taussig anastomosis, and 3 (7.9%) patients had undergone pulmonary artery repair before Fontan surgery. The mean age at the time of Fontan completion was 7.5 ± 3 years. Thirty-three (86.8%) patients had extracardiac Fontan tube, two (5.2%) patients each had lateral and intracardiac Fontan tube, and one (2.6%) patient had an atriopulmonary connection without tube.

### Cardiac MRI Findings

**Fig. 1:**
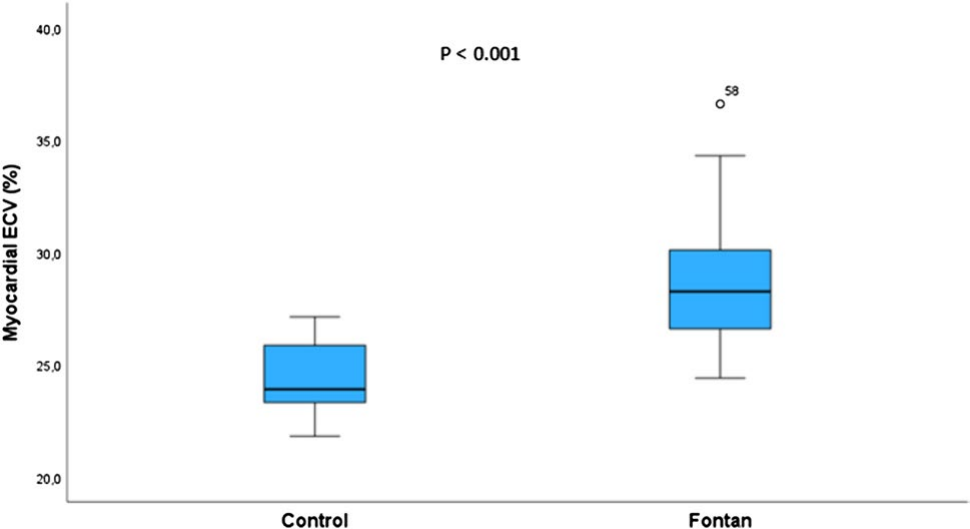
Myocardial extracellular volume (ECV) in controls and in patients with Fontan circulation

**Fig. 2:**
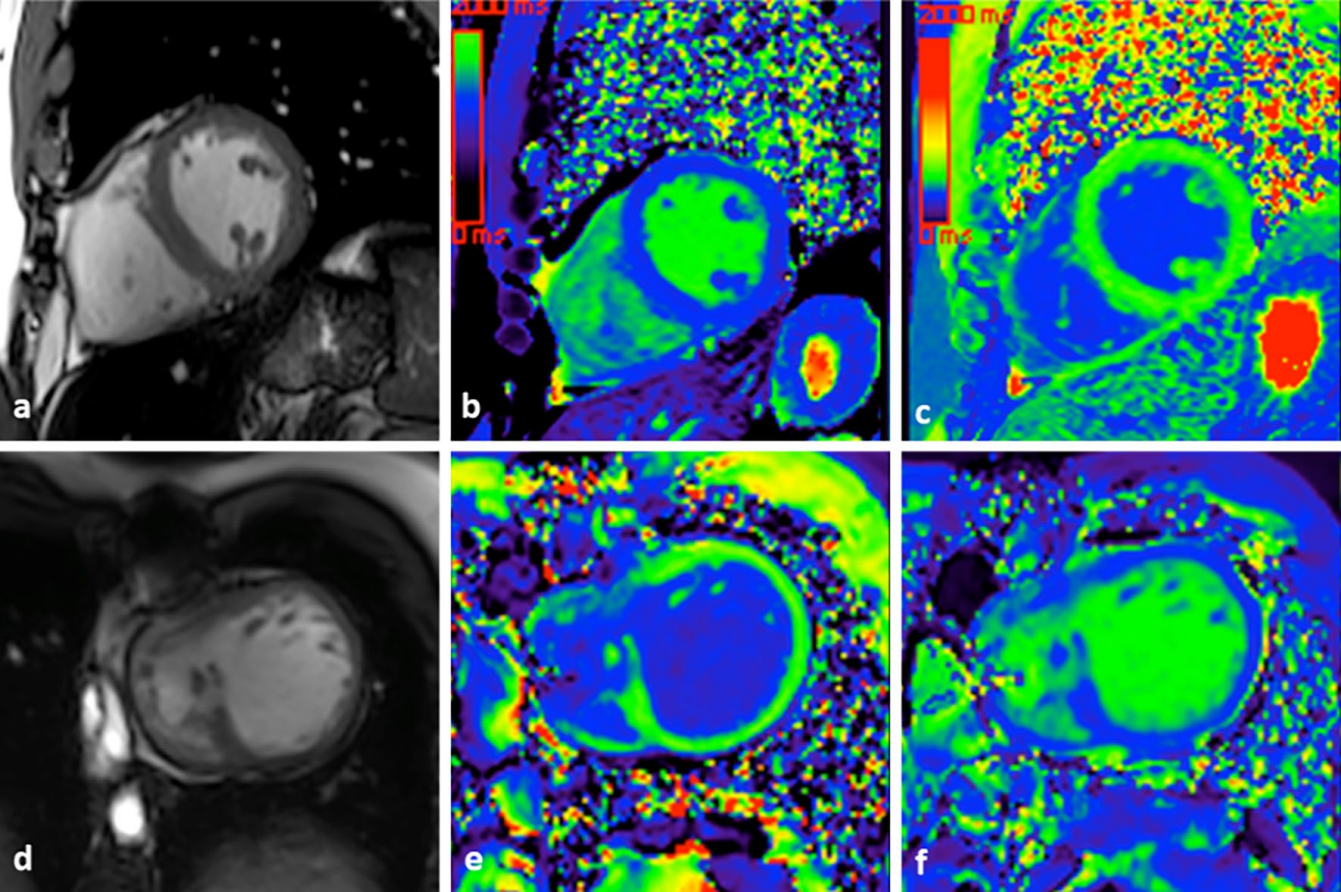
Cardiac short-axis anatomical (**a**, **d**), native (**b**, **e**), and post-contrast T1 mapping (**c**, **f**) sequences. Control subject (**a**–**c**), with native T1 value = 1206 ms and extracellular volume = 22.2%, and patient with tricuspid atresia and Fontan surgery (**d**–**f**) with native T1 value = 1394 ms and extracellular volume = 32.3%

**Fig. 3:**
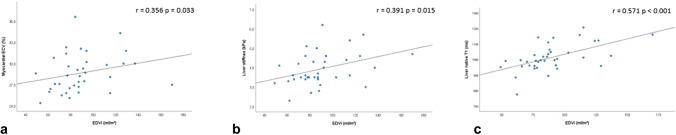
Linear scatterplots show positive correlations between ventricular indexed end-diastolic volume (EDVi) and: myocardial ECV (**a**), MRE-derived liver stiffness (**b**), and native liver T1 (**c**)

MRI examinations were conducted a mean of 8.5 ± 4.8 years after Fontan surgery. Relative to controls, the mean indexed ventricular end-diastolic volume (EDVi) was higher in patients with Fontan (89.6 ± 24.7 vs. 67.7 ± 12.1 ml/m^2^; p < 0.001), and the mean ventricular EF was lower in patients with Fontan (56% ± 8.7 vs. 72.5% ± 4.4; p < 0.001). The mean native T1 value (1242.5 ± 63.6 vs. 1208.5 ± 29.2 ms; *p* = 0.018) and the mean ECV (28.6% ± 2.7% vs. 24.4 ± 1.7; p < 0.001; Figs. 1 and 2) were higher in patients with Fontan. LGE was detected in six (16.2%) cases and no controls. LGE distribution was subendocardial in three patients, mesocardium in two and within the surgical scar in one. MRI parameters from patients with Fontan physiology and controls are depicted in Table 3. There was a positive correlation between myocardial ECV and EDVi (*r* = 0.356; *p* = 0.033; Fig. 3a) and a negative correlation between ejection fraction and weight (*r* = − 0.381; *p* = 0.018). There was no correlation between myocardial ECV and weight, age, or ejection fraction.

**Table 3:** MRI parameters

MRI parameters	Fontan (*n* = 38)			Controls (*n* = 23)			*p* value*
	Mean	Median	SD	Mean	Median	SD	
EDVi, ml/m^2^	89.6	86.5	24.7	67.7	67.0	12.1	< 0.001
ESVi, ml/m^2^	39.3	35.0	15.2	21.1	19.0	5.1	< 0.001
EF, %	56.0	56.0	8.7	72.5	73.0	4.4	< 0.001
Native cardiac T1 mapping	1242.5	1237.0	63.6	1208.5	1202.0	29.2	0.018
Myocardial ECV, %	28.6	28.2	2.7	24.4	23.9	1.7	< 0.001
Native liver T1 mapping	1013.6	996.0	86.0	780.8	775.0	71.0	< 0.001
Liver ECV, %	43.9	43.5	4.2	28.6	28.7	3.3	< 0.001
Liver stiffness^a^, kPa	3.93	3.60	0.84	2.17	2.10	0.45	< 0.001

*MRI* magnetic resonance imaging, *EDVi* systemic indexed ventricular end-diastolic volume, *ESVi* indexed ventricular end-systolic volume, *EF* ejection fraction; *ECV* extracellular volume, *GRE* gradient echo liver stiffness

*Mann–Whitney test

^a^For controls (*n* = 8)

### Liver MRI Findings

Hepatomegaly was detected in 97% of cases and additional signs of chronic liver disease, such as parenchymal heterogeneity and irregular liver contour, were detected in 71% of cases. Liver nodules with arterial hyperenhancement suggestive of focal nodular hyperplasia-like lesions were found in 23.6% of the patients, and inferior vena cava was dilated in 63.1%. One patient exhibited abdominal venous collaterals and two had ascites. No image was suggestive of hepatocellular carcinoma.

**Fig. 4:**
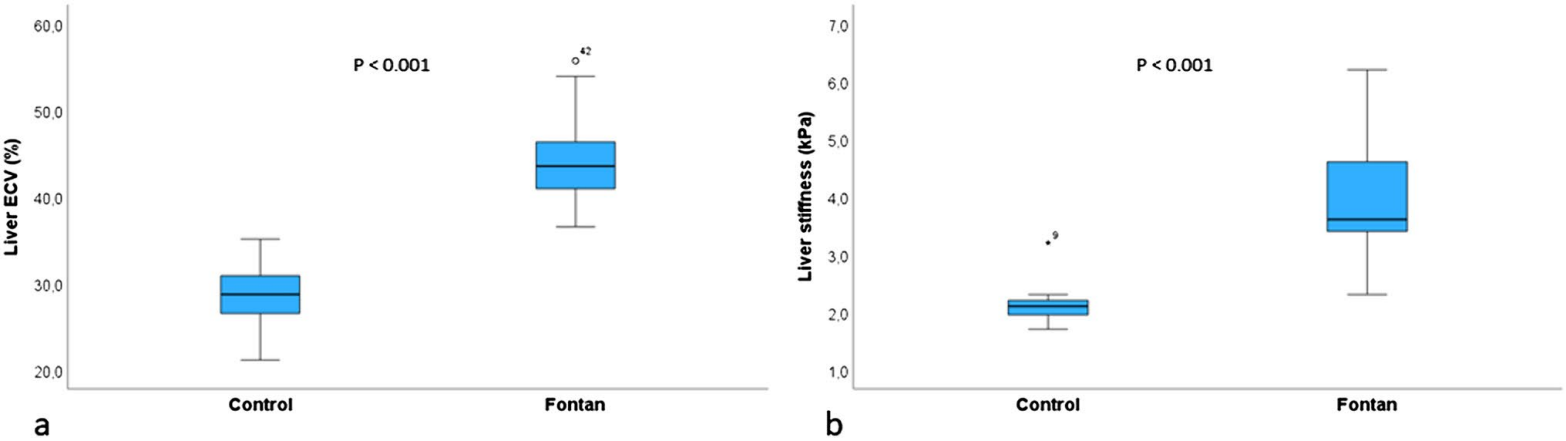
Liver extracellular volume (**a**) and MRE-derived liver stiffness (**b**) in controls and in patients with Fontan circulation

**Fig. 5:**
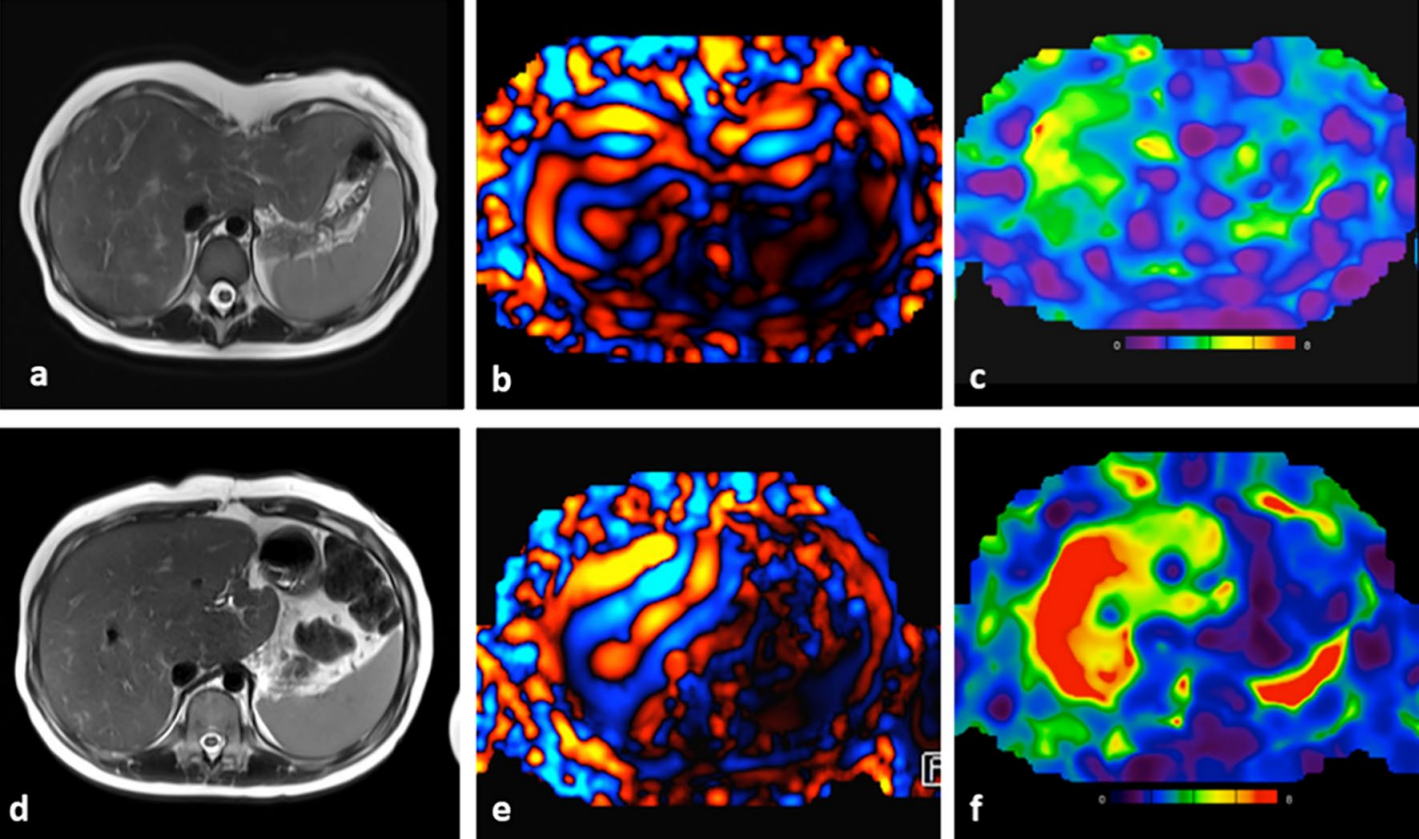
Liver axial haste anatomical images (**a**, **d**), MRE wave images (**b**, **e**), and color elastogram maps (**c**, **f**). Control subject (**a**–**c**) shows thin waves on MRE and normal liver stiffness values (2.1 kPa). Patient with Fontan physiology (**d**–**f**) with thick waves on MRE and elevated liver stiffness values (5.7 kPa﻿)

**Fig. 6:**
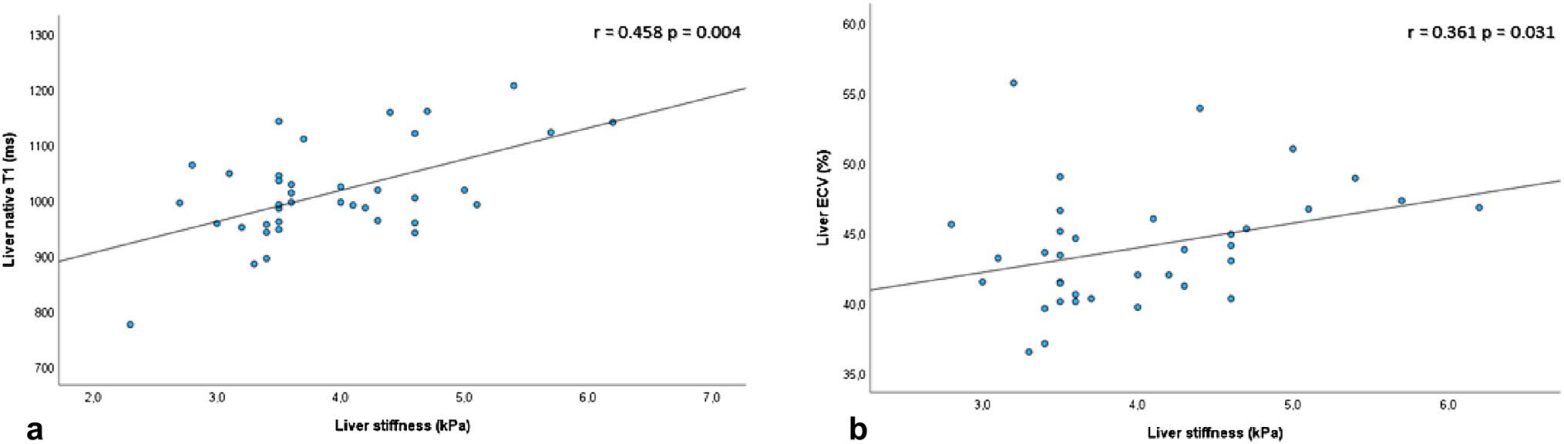
Linear scatterplots show positive correlations between MRE-derived liver stiffness with native liver T1 (**a**) and liver ECV (**b**)

Relative to controls, liver native T1 (1013.6 ± 86.0 vs. 780.8 ± 71.0 ms; *p* < 0.001), liver ECV (43.9% ± 4.2 vs. 28.6 ± 3.3; *p* < 0.001), and liver stiffness (3.93 ± 0.84 vs. 2.17 ± 0.45 kPa; *p* < 0.001; Figs. 4 and 5) were higher in patients with Fontan (Table 3). In patients with Fontan, liver stiffness correlated with weight (*r* = 0.364; *p* = 0.025), age (*r *= 0.376; *p* = 0.02), native liver T1 (*r* = 0.458; *p* = 0.004; Fig. 6a), and liver ECV (*r* = 0.361; *p* = 0.031; Fig. 6b). Liver stiffness was not significantly higher in patients with liver focal nodular hyperplasia-like lesions compared to patients with no focal lesions (4.4 vs. 3.8; *p* = 0.054).

### Associations Between Cardiac and Liver MRI Parameters

There was no correlation between myocardial ECV and liver stiffness or liver ECV. EDVi correlated with liver stiffness (*r* = 0.391; *p* = 0.015; Fig. 3b) and with native liver T1 (*r* = 0.571; *p* < 0.001; Fig. 3c). EDVi, EF, LGE, myocardial native T1 value, myocardial ECV were not associated with the presence of liver nodules.

## Discussion

Although the long-term consequences of the Fontan procedure are a growing concern due to circuit failure [5, 6], most patients in this cross-sectional study were in NYHA functional class I and had preserved ventricular function. Patients with Fontan had increased myocardial and liver native T1 and ECV, and increased MRE derived liver stiffness compared to controls. Increased end-diastolic ventricular volume correlated with cardiac and liver fibrosis and with liver stiffness.

Increased ECV has been associated with the histological finding of diffuse myocardial fibrosis with an increase of collagen in the interstitial space [26, 27]. Myocardial ECV has been used as an early prognostic marker for various cardiac diseases [27, 28], and our findings of increased native T1 and ECV in patients with Fontan circulation are in line with previous studies [29–31]. Interstitial fibrosis detected by increased ECV has been associated with decline of systolic ventricular function and increased ventricular stiffness in individuals with ischemic and non-ischemic heart diseases [10, 27, 32–34]. Nevertheless, we found no correlation between elevated ECV and lower ejection fraction, suggesting that the decline in ejection fraction may occur at a later stage of the disease, as reported Kato et al. [29] and Anderson et al. [35] and emphasizing that ECV is an early prognostic biomarker.

In the present study, there was a positive correlation between myocardial ECV and EDVi that may reflect the influence of chronic volume load in fibrotic remodeling, and reinforces the correlation between ECV and end-diastolic pressure reported by Alsaied et al. [30]. Ventricular remodeling has been associated with worse outcomes in these patients: Rathod et al. [36] determined that EDVi > 125 ml/m^2^ predict adverse events, and reported that progressive increases in indexed volumes have been associated with the onset of ventricular dysfunction in patients with Fontan circulation.

Relative to healthy controls, patients with Fontan exhibited higher liver native T1, ECV, and stiffness, with a positive correlation between liver ECV and stiffness measured by MRE. The universal presence of liver fibrosis after the Fontan operation has been reported in previous studies, and fibrotic progression has been associated with duration of exposure to the Fontan circulation [37]. In line with our findings, increased liver native T1 and ECV in Fontan patients have been reported by de Lange et al. [38] and Ramachandran et al. [39]. These findings may reflect the persistence of hepatic congestion years after Fontan surgery, with an associated fibrotic process that indicates progression to Fontan-associated liver disease.

Even though increased liver stiffness assessed by MRE does not allow us to differentiate congestion from fibrosis, a positive correlation between liver stiffness and fibrosis on biopsy has been reported recently in Fontan patients [40]. T1 and ECV may be less sensitive to hepatic flow changes, compared to MRE; however, it is still unclear whether liver native T1 mapping and ECV can help to differentiate between these conditions [30]. Liver biopsy remains the gold standard for diagnosis of liver fibrosis in many situations [41–43]. However, this technique is invasive and may be affected by sampling variability. New MRI parameters, such as liver stiffness, native T1 and ECV are promising tools to assess and monitor the hepatic status in patients with Fontan circulation to avoid progression to liver cirrhosis [44, 45].

An increased prevalence of nodular hyperplasia-like lesions was demonstrated in the present study. Although we could not show an association between these lesions and increased liver native T1 and stiffness, they probably reflect the progression of the liver disease. Previous studies corroborate that liver nodules are frequent in Fontan patients. Téllez et al. [46] reports that some liver nodules may go unnoticed on ultrasound screening. There is an increased risk of hepatocellular carcinoma in these patients [47–49].

End diastolic ventricular volume was associated with liver stiffness in our work, which confirms the findings reported by Alsaied et al. [30] and indicates that cardiac overload may be responsible for a greater passive venous congestion, a possible substrate to the fibrotic process in the liver. Ventricular remodeling also correlated with both myocardial ECV and liver native T1, suggesting that patients with increased volume overload have a higher risk to develop cardiac and liver fibrosis. Although Beigh et al. [31] reported a correlation between liver and cardiac native T1, they did not find an association between these parameters and ventricular remodeling. On the other hand, we could not show a correlation between cardiac ECV and liver ECV or stiffness.

Myocardial dysfunction and Fontan-associated liver disease with cirrhosis are expected outcomes for patients with failing Fontan and many centers offer a combined heart-liver transplantation [50, 51]. The use of multiparametric MRI to monitor the fibrotic process in the liver and myocardium is crucial to predict the progression to irreversible cardiac and liver fibrosis and could provide new insights to improve treatment strategies.

The present study has some limitations. The sample was relatively small, due to the rarity of the disease, and we did not perform liver biopsy in our patients. However, MRE has been validated as an effective method to assess liver stiffness and prior research has indicated an association between hepatic stiffness and histological fibrosis in different diseases, including patients with Fontan [40, 52, 53]. Although we did not have age-matched controls; we could demonstrate increased myocardial and liver fibrosis in patients despite their younger age compared to controls. The increased age of the control group did not impact our findings considering that ECV and liver stiffness increase with age so the values in age-matched controls would be even lower [54, 55].

Multiparametric MRI is a valuable tool for the comprehensive assessment of liver and cardiac fibrosis in these patients. It is critical to improve the understanding of the progression of myocardial fibrosis and dysfunction and liver disease in patients who have undergone Fontan procedures to prevent the need of combined liver and heart transplantation.

## Conclusion

The increased prevalence of myocardial and liver fibrosis, and the increased liver stiffness in patients with univentricular physiology justify the value of multiparametric MRI for the combined assessment of cardiac and liver abnormalities in these patients. Emerging MRI modalities, such as MRI T1 mapping, ECV calculation, and MRE are useful to assess the progression of cardiac and liver disease and to improve treatment strategies in patients who have undergone Fontan procedures.

## Data Availability

The data presented in this study are available on request from the corresponding author.

## Abbreviations

EF: Ejection fraction
LGE: Late gadolinium enhancement
EDVi: Indexed end diastolic volume
FALD: Fontan-associated liver disease
LGE: Late gadolinium enhancement
MRE: Magnetic resonance elastography
MRI: Magnetic resonance image
